# GNNMF: a multi-view graph neural network for ATAC-seq motif finding

**DOI:** 10.1186/s12864-024-10218-0

**Published:** 2024-03-21

**Authors:** Shuangquan Zhang, Xiaotian Wu, Zhichao Lian, Chunman Zuo, Yan Wang

**Affiliations:** 1 School of Cyber Science and Engineering, Nanjing University of Science and Technology, Nanjing, 210094 China; 2 School of Artificial Intelligence, Jilin University, Changchun, 130012 China; 3 Institute of Artificial Intelligence, Donghua University, Shanghai, 201620 China; 4 Key Laboratory of Symbol Computation and Knowledge Engineering of Ministry of Education, College of Computer Science and Technology, Jilin University, Changchun, 130012 China

**Keywords:** Graph neural network, Coexisting probability, ATAC-seq motifs, Multi-view heterogeneous graph

## Abstract

**Background:**

The Assay for Transposase-Accessible Chromatin using sequencing (ATAC-seq) utilizes the Transposase Tn5 to probe open chromatic, which simultaneously reveals multiple transcription factor binding sites (TFBSs) compared to traditional technologies. Deep learning (DL) technology, including convolutional neural networks (CNNs), has successfully found motifs from ATAC-seq data. Due to the limitation of the width of convolutional kernels, the existing models only find motifs with fixed lengths. A Graph neural network (GNN) can work on non-Euclidean data, which has the potential to find ATAC-seq motifs with different lengths. However, the existing GNN models ignored the relationships among ATAC-seq sequences, and their parameter settings should be improved.

**Results:**

In this study, we proposed a novel GNN model named GNNMF to find ATAC-seq motifs via GNN and background coexisting probability. Our experiment has been conducted on 200 human datasets and 80 mouse datasets, demonstrated that GNNMF has improved the area of eight metrics radar scores of 4.92% and 6.81% respectively, and found more motifs than did the existing models.

**Conclusions:**

In this study, we developed a novel model named GNNMF for finding multiple ATAC-seq motifs. GNNMF built a multi-view heterogeneous graph by using ATAC-seq sequences, and utilized background coexisting probability and the iterloss to find different lengths of ATAC-seq motifs and optimize the parameter sets. Compared to existing models, GNNMF achieved the best performance on TFBS prediction and ATAC-seq motif finding, which demonstrates that our improvement is available for ATAC-seq motif finding.

**Supplementary Information:**

The online version contains supplementary material available at 10.1186/s12864-024-10218-0.

## Background

Transcription factors (TFs) are proteins that can bind to DNA sequences and play a crucial role in gene-regulated networks [1], cell cycle regulation [2], and human diseases [3]. The region where TFs bind to DNA sequences is called transcription factor binding sites (TFBSs), which are short and conserved DNA fragments [4]. Transcription regulation is carried out by the interplay between TFs and TFBSs in DNA sequences, and identifying TFBSs aids us to reveal functions of TFs and the cause of human diseases [5]. The aligned TFBSs of the same TF can be defined as a regulatory DNA motif, which can be represented by a position weight matrix (PWM) [6]. Assays for Transposase-Accessible Chromatin using sequencing (ATAC-seq) can probe open chromatin via Tn5, which is an effective method for locating TFBSs at a genome-wide level [7, 8]. Compared to Chromatin immunoprecipitation sequencing (ChIP-seq) and DNase I hypersensitive sites sequencing (DNase-seq) technologies, ATAC-seq can reveal more kinds of transcription factor binding regions [9]. An ATAC-seq footprint is a fragment of a DNA sequence that has not been cleaved due to the transcription factors binding. TOBIAS and Hint-ATAC are feasible tools for identifying ATAC-seq footprints, TOBIAS utilizes bias correction [10] and footprinting scores to locate footprints, and Hint-ATAC employs the hidden Markov model to locate footprints [11]. ATAC-seq footprints have been applied to TF network prediction [11], comparison of TF activity [12], identification of TFs enriched in peripheral blood mononuclear cells (PBMC)-specific peaks [13], and ATAC-seq motifs finding [14].

There are several special tools for ATAC-seq motif scanning, such as TRACE, chromVAR, SnapATAC [15–17]. They scan input sequences by using known motifs, which limits their ability to find ATAC-seq motifs. DL technology, including convolutional neural networks (CNNs) and the recurrent neural networks has achieved successes in protein-protein networks, gene-regulated networks, and motifs finding [18–20]. ATAC-seq motif finding includes two key steps: the first step is to predict an ATAC-seq sequence that contains TFBSs, *i.e.* TFBS prediction, and the second step is to find ATAC-seq motifs. Existing models such as scFAN, FactorNet, and DeepATAC employ CNNs to predict TFBSs and find motifs from ATAC-seq data [21, 22]. These models utilized convolutional kernels of CNNs to scan ATAC-seq sequences, and a fully connected layer to predict TFBSs. Due to the limitation of the width  of convolutional kernels, these models only find motifs with fixed lengths. Moreover, these existing models do not consider the coexisting probability of TFBSs in an input sequence. In a related line of research, the graph neural network (GNN) can learn the key message from the graph-structured data [23]. GNNs can learn the embeddings of nodes via their neighboring nodes, and keep the connection of the graph unchanged and nodes’ embedding can also enable graph-based explanation and reasoning. GNNs work on non-Euclidean structured data and have been shown to perform well on molecular structures [24], protein-protein networks [25], gene-gene networks [26], ATAC-seq motif finding [14], *etc*. MMGraph is an ATAC-seq motif predictor based on GNN and coexisting probability to find multiple motifs [14]. MMGraph utilizes ATAC-seq footprints to build a multi-view heterogeneous graph by defining coexisting, Hamming, jaccard, and inclusive edges. Compared with CNN-based models, MMGraph utilizes ATAC-seq footprints to build the multi-view heterogeneous graph and employs the coexisting probability to find multiple ATAC-seq motifs of different lengths. However, MMGraph still has certain defects. MMGraph sets the coexistence probability threshold to 0.5, which needs to be optimized. MMGraph uses only Hamming distance and coexisting probability to measure the relationships between k-mers, which ignored the correlation between k-mers.

To address the above issues, this study developed a novel model called GNNMF to find ATAC-seq motifs and predict TFBSs. GNNMF is based on MMGraph and has three improved aspects: built a multi-view heterogonous graph, which contains four kinds of edges (coexisting edges, similarity edges, jaccard edges, and inclusive edges) and two types of nodes (k-mer nodes and sequence nodes); defined the iterloss function to improve TFBS prediction accuracy; optimized the threshold of coexisting probability via defining coexisting probability between k-mer nodes in negative sequences. We tested the effectiveness of the GNNMF model on 200 human ATAC-seq datasets and 80 mouse ATAC-seq datasets across nine evaluation metrics, including the area of eight metrics radar (AEMR), precision, recall, F1_score, accuracy (ACC), specificity, the Matthews correlation coefficient (MCC), the area under the receiver operating characteristic curve (AUC), and the area under the precision-recall curve (PRC). According to the experimental results, GNNMF improved the ACC, MCC, and AUC by 9.6%, 18.2%, and 2.9%, respectively, on 200 human ATAC-seq datasets and by 2.36%, 6.35%, and 3.01%, respectively, on 80 mouse ATAC-seq datasets. In this study, AEMR is an overall score for evaluating the model’s ability to predict TFBSs. GNNMF improved the AEMR by 4.92% and 6.81% on 200 human and 80 mouse ATAC-seq datasets, respectively. Moreover, among all the models, GNNMF find the most 385 and 662 significant motifs from human ATAC-seq data and mouse ATAC-seq data , respectively.

## Methods

### Data processing

The 280 ATAC-seq datasets, including 200 human ATAC-seq datasets and 80 mouse ATAC-seq datasets, were randomly downloaded from the ENCODE project (Supplementary Table S1). To obtain corrected footprints, footprints of each dataset are obtained via TOBIAS and Hint-ATAC tools with default parameters [11, 12]. All footprints are ranked in descending order by their scores. Then the top 1500 footprints are used to intersect with the footprints that Hint-ATAC obtains, and the intersected footprints of each dataset are applied to the following analysis.

Each intersected footprint is pruned with 101 bps around its center by the bedtools [27], which is set as a positive sequence. The TFBS prediction is a binary classification, so we shuffle all bases within a positive sequence as a negative sequence [28]. This paper gives a positive sequence a label of ‘1’, and gives a negative sequence a label of ‘0’. Thus, we can obtain an ATAC-seq sequence set $S$ that includes $n$ sequences.

For each dataset, 80% of $S$ is set as training data, 10% of $S$ is set as validation data, and the remaining $S$ is set as testing data. Then, the sequence $s( \cdot )$ ($s( \cdot ) \in S$) is trimmed into k-mers $k( \cdot )$ by the step of one base to obtain a k-mer set $Ks( \cdot )$($lenk = length(k( \cdot ))$). $s( \cdot )$ and k-mer $k( \cdot )$ will be utilized to build the multi-view heterogeneous graph $G$, where $s( \cdot )$ and $k( \cdot )$ are nodes.

### Building the multi-view heterogeneous graph

**Fig. 1:**
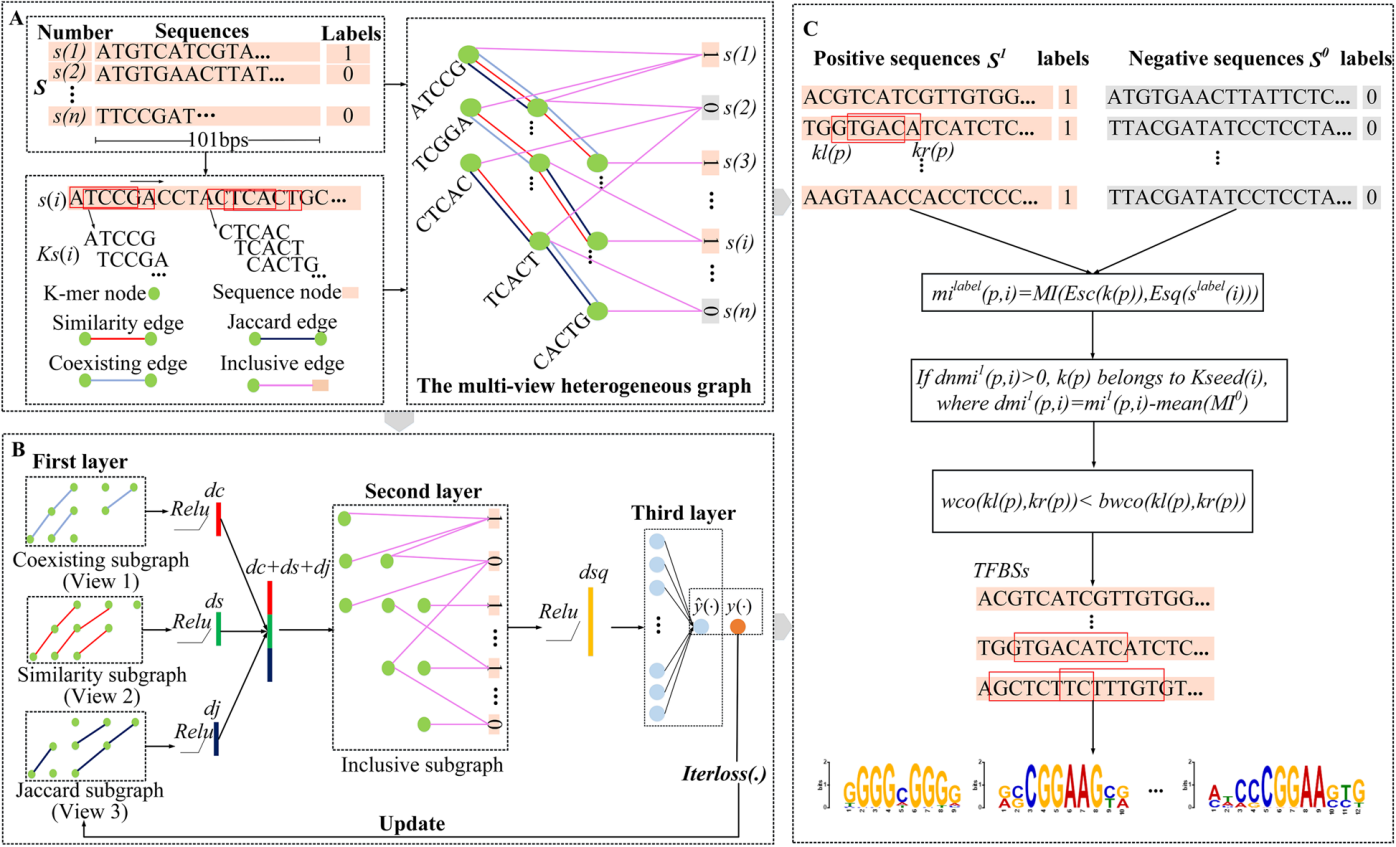
The GNNMF framework

The multi-view heterogeneous graph $G$ contains two kinds of nodes (sequence node $s( \cdot )$ and k-mer node $k( \cdot )$), and four types of edges (coexisting edge, similarity edge, Jaccard edge and inclusive edge) (Fig. 1A). Coexisting edges (view 1) represent coexisting relationships between k-mer nodes in a sequence, and the weights of coexisting edges are calculated by the coexisting probability [14]. Similarity edges (view 2) represent relations between k-mer nodes and the weights of similarity edges are measured by Hamming distances [29]. Jaccard edges (view 3) represent relationships between k-mer nodes, and the weights of Jaccard edges are measured by the Jaccard correlation coefficient. Inclusive edges represent inclusive relations between a given sequence $s( \cdot )$ and its constituent k-mers, and weights of inclusive edges are measured by TF-IDF (term frequency-inverse document frequency) [30].

We let $Ks(i)$ be a k-mer set that contains all unique $k( \cdot )$ within $s(i)$. $K$ is an $m$ k-mer set that contains all unique $k( \cdot )$ in all $Ks(i)$ of $S$, where $m$ is the total number of unique $k( \cdot )$ within $S$. Four edge types are defined to build $G$, where $s( \cdot )$ and $k( \cdot )$ are nodes.

Coexisting edges measure the coexisting probability between $k( \cdot )$ nodes. The coexisting edge weight between two k-mers $k(p)$ and $k(j)$ is calculated by Formula (1):

1 $$\begin{gathered} wco(p,j) = wco(j,p) = {\kern 1pt} {\kern 1pt} - \log (\frac{Q(k(p),k(j))}{{P(k(p))P(k(j))}}){\kern 1pt} {\kern 1pt} {\kern 1pt} {\kern 1pt} {\kern 1pt} {\kern 1pt} {\kern 1pt} {\kern 1pt} if{\kern 1pt} {\kern 1pt} {\kern 1pt} nums(k(p),k(j)) > 0 \hfill \\ {\kern 1pt} {\kern 1pt} {\kern 1pt} {\kern 1pt} {\kern 1pt} {\kern 1pt} {\kern 1pt} {\kern 1pt} {\kern 1pt} {\kern 1pt} {\kern 1pt} {\kern 1pt} {\kern 1pt} {\kern 1pt} {\kern 1pt} {\kern 1pt} {\kern 1pt} {\kern 1pt} {\kern 1pt} {\kern 1pt} {\kern 1pt} {\kern 1pt} {\kern 1pt} {\kern 1pt} {\kern 1pt} {\kern 1pt} {\kern 1pt} {\kern 1pt} {\kern 1pt} {\kern 1pt} {\kern 1pt} {\kern 1pt} {\kern 1pt} {\kern 1pt} P(k(p)) = \frac{{num(k(p{)})}}{n},{\kern 1pt} {\kern 1pt} Q(k(p),k{\text{(j)}}) = \frac{nums(k(p),k(j))}{n} \hfill \\ \end{gathered}$$

where $Wco$ represents a $m \times m$ coexisting weight matrix, $num(k( \cdot ))$ represents the total number of $Ks( \cdot )$ that contain $k( \cdot )$, and $nums(k(p),k(j))$ is the total number of $Ks( \cdot )$ that contain both $k(p)$ and $k(j)$.

Similarity edges measure mismatches between $k( \cdot )$ nodes using the Hamming distance [31]. The similarity edge weight between $k(p)$ and $k(j)$ is calculated by Formula (2):

2 $$wsim(p,j) = wsim(j,p) = Hamming(k(p),k(j))$$

where $Wsim$ represents a $m \times m$ similarity weight matrix,${\kern 1pt} {\kern 1pt} p{\kern 1pt} ,j \in [1,...,m]$. $Ham\min g( \cdot , \cdot )$ represents the Hamming distance function.

Jaccard edges measure the correlation between $k( \cdot )$ nodes using the Jaccard correlation coefficient. The Jaccard edge weight between two nodes $k(p)$ and $k(j)$ is calculated by Formula (3):

3 $$wjac(p,j) = \frac{|Sk(k(p)) \cap Sk(k(j))|}{{|Sk(k(p)) \cup Sk(k(j))|}}$$

where $Sk( \cdot )$ presents a sequence set that contains all sequences, including $k( \cdot )$. $Wjac$ represents a $m \times m$ similarity weight matrix, ${\kern 1pt} p{\kern 1pt} ,j \in [1,...,m]$.

Inclusive edges measure the dependency degree between $s( \cdot )$ and $k( \cdot )$. The inclusive edge weight between $s( \cdot )$ and $k( \cdot )$ is calculated by transferring the concept of the term frequency-inverse document frequency (TF-IDF) [32] to Formula (4):

4 $$winclu(p,i) = tf(k(p),s(i)) \times \log (\frac{n}{num(k(p))}){\kern 1pt} {\kern 1pt} {\kern 1pt} {\kern 1pt} {\kern 1pt} {\kern 1pt} if{\kern 1pt} {\kern 1pt} k(p) \in Ks(i)$$

where $Winclu$ represents an $m \times n$ inclusive weight matrix,$tf(k(p),s(i))$ is the number of $k(p)$ existing in $s(i)$, $i \in [1,...,n]$, and $p \in [1,...,m]$.

### GNNMF overview

In this study, we developed a three-layer GNN model named GNNMF to find ATAC-seq motifs. GNNMF decomposes the multi-view heterogeneous graph $G$ into four subgraphs, *i.e.* a coexisting subgraph, similarity subgraph, Jaccard subgraph, and inclusive subgraph (Fig. 1B). The first layer of GNNMF is utilized to learn the embedding of $k( \cdot )$ as $Ek(k( \cdot )) \in {\mathbb{R}}^{dc + ds + dj}$ where $dc$, $ds$ and $dj$ are the embedding dimensions of $Esim(k( \cdot ))$($Esim(k( \cdot )) \in {\mathbb{R}}^{dc}$), $Eco(k( \cdot ))$($Eco(k( \cdot )) \in {\mathbb{R}}^{ds}$) and $Ejac(k( \cdot ))$ ($Ejac(k( \cdot )) \in {\mathbb{R}}^{dj}$) from the coexisting subgraph, similarity subgraph and Jaccard subgraph, respectively. The second layer learns the embedding of $s( \cdot )$ as $Es(s( \cdot ))$ ($Es(s( \cdot )) \in {\mathbb{R}}^{dsq}$) from the inclusive subgraph, where $dsq$ is the dimension of $Es(s( \cdot ))$. The third layer is a fully connected layer, which is used to predict TFBSs.

We normalize $Wco$, $Wsim$ and $Wjac$ as the initial embedding of $k( \cdot )$ via Formula (5).

5 $$\begin{gathered} Ect(k(p)) = (\sum\limits_{j = 1}^{m} {Wco(p,j))^{ - 1} \times Wco(p,:)} \hfill \\ Est(k(p)) = (\sum\limits_{j = 1}^{m} {Wsim(p,j))^{ - 1} \times Wsim(p,:)} \hfill \\ Ejt(k(p)) = (\sum\limits_{j = 1}^{m} {Wjac(p,j))^{ - 1} \times Wjac(p,:)} \hfill \\ \end{gathered}$$

where $Ect(k(p))$ ($Ect(k(p)) \in {\mathbb{R}}^{dc}$) is the initial embedding of $k(p)$, based on $Wco$;$Est(k(p))$($Est(k(p)) \in {\mathbb{R}}^{ds}$) is the initial embedding of $k(p)$, based on $Wsim$;$Ejt(k(p))$($Ejt(k(p)) \in {\mathbb{R}}^{dj}$) is the initial embedding of $k(p)$, based on $Wjac$.

GNNMF learns the embedding of $k(p)$ as $Eco(k(p))$ from the coexisting subgraph, and $Eco(k(p))$ is calculated by Formula (7).

6 $$eco(k(p)) = Ect(k(p)) \times Wco \times W^{co}$$

7 $$Eco(k(p)) = {\text{Re}} LU(eco(k(p)))$$

where $W^{co}$($W^{co} \in {\mathbb{R}}^{m \times dc}$) is a training weight matrix, which is randomly initialized. ${\text{Re}} LU( \cdot )$ represents the rectified linear unit, which is an activation function.

GNNMF learns $Esim(k(p))$ from the similarity subgraph via Formula (9).

8 $$esim(k(p)) = Est(k(p)) \times Wsim \times W^{sim}$$

9 $$Esim(k(p)) = {\text{Re}} LU(esim(k(p)))$$

where $Esim(k(p))$ is the embedding of $k(p)$ in the similarity subgraph. $W^{sim}$($W^{sim} \in {\mathbb{R}}^{m \times ds}$) is a training weight matrix, which is randomly initialized.

GNNMF learns the embedding of $k(p)$ as $Ejac(k(p))$ from the Jaccard subgraph, and $Ejac(k(p))$ is calculated by Formula (11).

10 $$ejac(k(p)) = Ejt(k(p)) \times Wjac \times W^{jac}$$

11 $$Ejac(k(p)) = {\text{Re}} LU(ejac(k(p)))$$

where $W^{jac}$($W^{jac} \in {\mathbb{R}}^{m \times dj}$) is a training weight matrix that is randomly initialized.

Then, $Esim(k( \cdot ))$, $Eco(k( \cdot ))$ and $Ejac(k( \cdot ))$ are concatenated as $Esc(k( \cdot ))$ (Formula (12)), which is fed into the second layer.

12 $$Esc(k(p)) = concatenate([Eco(k(p)),Esim(k(p)),Ejac(k(p))])$$

We stack $Esc(k(p))$ into a matrix $Msc$ ($Msc \in {\mathbb{R}}^{m \times (ds + dc + dj)}$). GNNMF learns the embedding of $s( \cdot )$ as $Esq(s( \cdot ))$ via the second layer, $Esq(s( \cdot ))$ is calculated by Formula (14).

13 $$esq(s(i)) = Winclu(:,i)^{T} \times Msc \times W^{inclu}$$

14 $$Esq(s(i)) = {\text{Re}} LU(esq(s(i)))$$

The third layer is a fully connected layer, which is used to predict TFBSs (Formula (16)).

15 $$\hat{y}(s(i)) = W(i,:) \times Esq(s(i){)} + b(i)$$

16 $$\hat{y}(s(i)) = sigmoid(y^{\prime}(s(i)))$$

where $W$($W \in {\mathbb{R}}^{n \times dsq}$) is the training weight matrix, which is randomly initialized. $b$ represents the bias, which is a $n \times 1$ vector, $sigmoid( \cdot )$ is a sigmoid function, and $\hat{y}(s(i))$ represents the predicted label of $s(i)$ by GNNMF.

The BCEloss is the binary cross-entropy loss, which is used to calculate the binary cross-entropy loss between the predicted probability and the true label. Based on BCEloss, we define the iterloss as the loss function of GNNMF [33]:

17 $$BCEloss(s(i)) = - [y(s(i)) \times \log \hat{y}(s(i)) + (1 - y(s(i))) \times \log (1 - \hat{y}(s(i)))]$$

18 $$Dloss(s(i))^{epoch - 1} = BCEloss(s(i))^{epoch - 1} \times sigmoid(BCEloss(s(i))^{epoch - 1} )$$

19 $${\text{i}} terloss(i) = Dloss(s(i))^{epoch - 1} + BCEloss(s(i))^{epoch}$$

where $y(s(i))$ represents the true label of sequence $s(i)$ (Formula (19)); $\hat{y}(s(i))$ represents the predicted label of sequence $s(i)$; and $epoch$ represents the iteration, $BCEloss(s(i))^{0} = 0$.

### Find multiple motifs via coexisting probability between k-mers

GNNMF calculates the mutual information (MI) $mi(p,i)$ between $k(p)$ and $s(i)$ by using $Esc(k( \cdot ))$ and $Esq(s( \cdot ))$ (Fig. 1C). This study divided $S$ into $S^{0}$ and $S^{1}$ by $label = 1/0$, as well as $n^{label}$, $m^{label}$, $s^{label} ( \cdot )$, $Ks^{label} ( \cdot )$ and $K^{label}$. GNNMF calculates the $MI^{label}$ matrix by $mi(p,i)$, where ${\kern 1pt} k(p) \in K^{label}$, $s(i) \in s^{label} ( \cdot )$, $i \in [1,...,n^{label} ]$. We define $mean(MI^{0} )$ as the background noise of $MI^{1}$ and calculate the denoised MI matrix $dnMI^{1}$, where $mean(MI^{0} ) = (\sum\limits_{p = 1}^{{m^{0} }} {\sum\limits_{i = 1}^{{n^{0} }} {mi^{0} (p,i)} )/(m^{0} \times n^{0} )}$ and $dnmi^{1} (p,i) = mi^{1} (p,i) - mean(MI^{0} )$. We define the k-mer seed set $Kseed(i)$ that contains all unique $k(p)$ in $s^{1} (i)$, when $dnmi^{1} (p,i) > 0$,$i \in [1,...,n^{1} ]$. Then, we locate the interval of $k(p)$ in $s^{1} (i)$ as $itk(p) = \left[ {strk(p),strk(p) + lenk - 1} \right]$, where $k(p) \in Kseed(i)$,$strk(p)$ is the starting position of $k(p)$ in $s^{1} (i)$. If multiple $k(p)$ exist in $s^{1} (i)$, there will be multiple $itk(p)$. We obtain two $k( \cdot )$ centered on $itk(p)$ as $kl(p) = [ck(p) - lenk + 1,ck(p)]$ and $kr(p) = [ck(p){ + }1,ck(p) + lenk{]}$, where $ck(p) = strk(p) + \left\lceil {(lenk - 1)/2} \right\rceil$ and $kl(p),kr(p) \in K$.

We calculate the coexisting probability $wco(kl(p),kr(j))$ and background probability $bwco(kl(p),kr(p))$ between $kl(p)$ and $kr(p)$, respectively. If $wco(kl(p),kr(p)) < bwco(kl(p),kr(p))$, which means that $kl(p)$ and $kr(p)$ are strongly related, GNNMF merges $kl(p)$ and $kr(p)$ to a TFBS as $tfbs(p) = [ck(p) - lenk{ + }1,ck(p) + lenk]$. For all $k( \cdot ) \in Kseed(i)$, we can find multiple candidates $tfbs(i)$ in $s^{1} (i)$. If multiple $tfbs(i)$ overlap, they are merged into a longer TFBS in $s^{1} (i)$. Finally, GNNMF can find multiple $tfbs( \cdot )$ with different lengths.

### Evaluation metrics

Our evaluation metrics include precision, recall, F1_score, ACC, specificity, MCC, AUC, PRC, and AEMR [34]. AEMR is the area of a radar chart, which can be generated that consists of eight equiangular spokes with each spoke representing one of the scores defined above [14]. The higher the AEMR score is, the better the performance of model for TFBS prediction.

All the evaluation metrics are calculated via the following formulas:

20 $$Precision = \frac{TP}{{TP + FN}}$$

21 $${\text{Re}} call = \frac{TP}{{TP + TN}}$$

22 $$\frac{2}{f1\_score} = \frac{1}{precision} + \frac{1}{recall}$$

23 $$Specificity = \frac{TN}{{FP + TN}}$$

24 $$ACC = \frac{TN + TP}{{FP + FN + TP + TN}}$$

25 $$MCC = \frac{TP \times TN - FP \times FN}{{\sqrt {(TP + FP) \times (FP + FN) \times (TN + FP) \times (TN + FN)} }}$$

where TP, TN, FP and FN represent the number of the true positive (TP), true negative (TN), false positive (FP), and false negative (FN), respectively.

The AUC is the area under the receiver operating characteristic curve, and the PRC is the area under the precision-recall curve, which is a value between 0 and 1.

26 $$O_{i,i + 1} = \frac{1}{2}R_{i} \times R_{i + 1} \times \sin (\frac{\pi }{4}){\kern 1pt} {\kern 1pt} {\kern 1pt} {\kern 1pt} {\kern 1pt} {\kern 1pt} {\kern 1pt} {\kern 1pt} {\kern 1pt} i = 1,...,8$$

27 $$\begin{gathered} R = [precision,recall,F1\_score,ACC,specificity,MCC,AUC,PRC,precision] \hfill \\ AEMR = sum(O_{i,i + 1} ){\kern 1pt} {\kern 1pt} {\kern 1pt} {\kern 1pt} {\kern 1pt} {\kern 1pt} {\kern 1pt} i = 1,...,8 \hfill \\ \end{gathered}$$

### Experiment settings

**Fig. 2:**
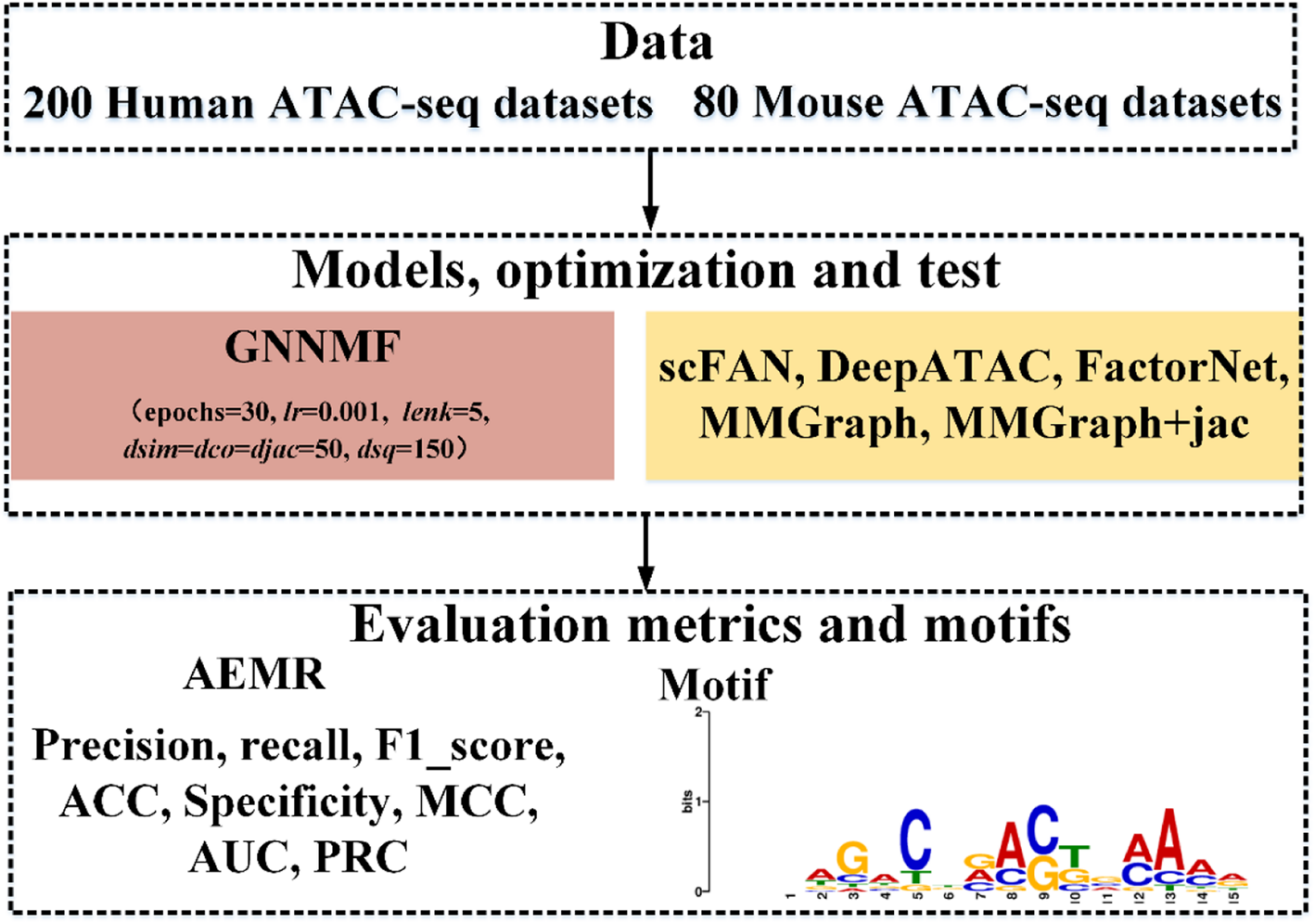
The workflow of the experimental setting

This study trained GNNMF for 30 epochs using the Adam optimizer [35] (Fig. 2). The hyperparameters of GNNMF included the learning rate, $dsim$, $dco$, $djac$, $dsq$ and $lenk$, and their ranges were tested in this study (Supplementary Table S2). We utilized the grid search to choose the optimal parameters and applied the combinations of those hyperparameters to GNNMF model. The AUC is used to measure the performance of GNNMF with different combinations on the testing set. The learning rate of GNNMF was set as 0.001 and was adjusted by the natural exponential decay with 0.001. When we trained GNNMF on the training set, the sequence nodes in validation set and the testing set were masked. The $dsim$, $dco$ and $djac$ were set as 50, and $dsq$ was set as 150 in our experiment. Considering the computational complexity and the performance of GNNMF, we set $lenk = 5$. The scFAN, DeepATAC, FactorNet, MMGraph, and MMGraph+jac models are used as comparison tools, where MMGraph+jac combines MMGraph and jaccard graph. The evaluation metrics, including precision, recall, F1_score, ACC, specificity, MCC, AUC, PRC, and AEMR, are utilized to evaluate models’ performance on TFBS prediction. To explain the GNNMF model, ATAC-seq motifs are used to show features that GNNMF learned. We matched the found motifs to motifs that the HOCOMOCO database contains via the TOMTOM tool.

## Results

### TFBS prediction

**Fig. 3:**
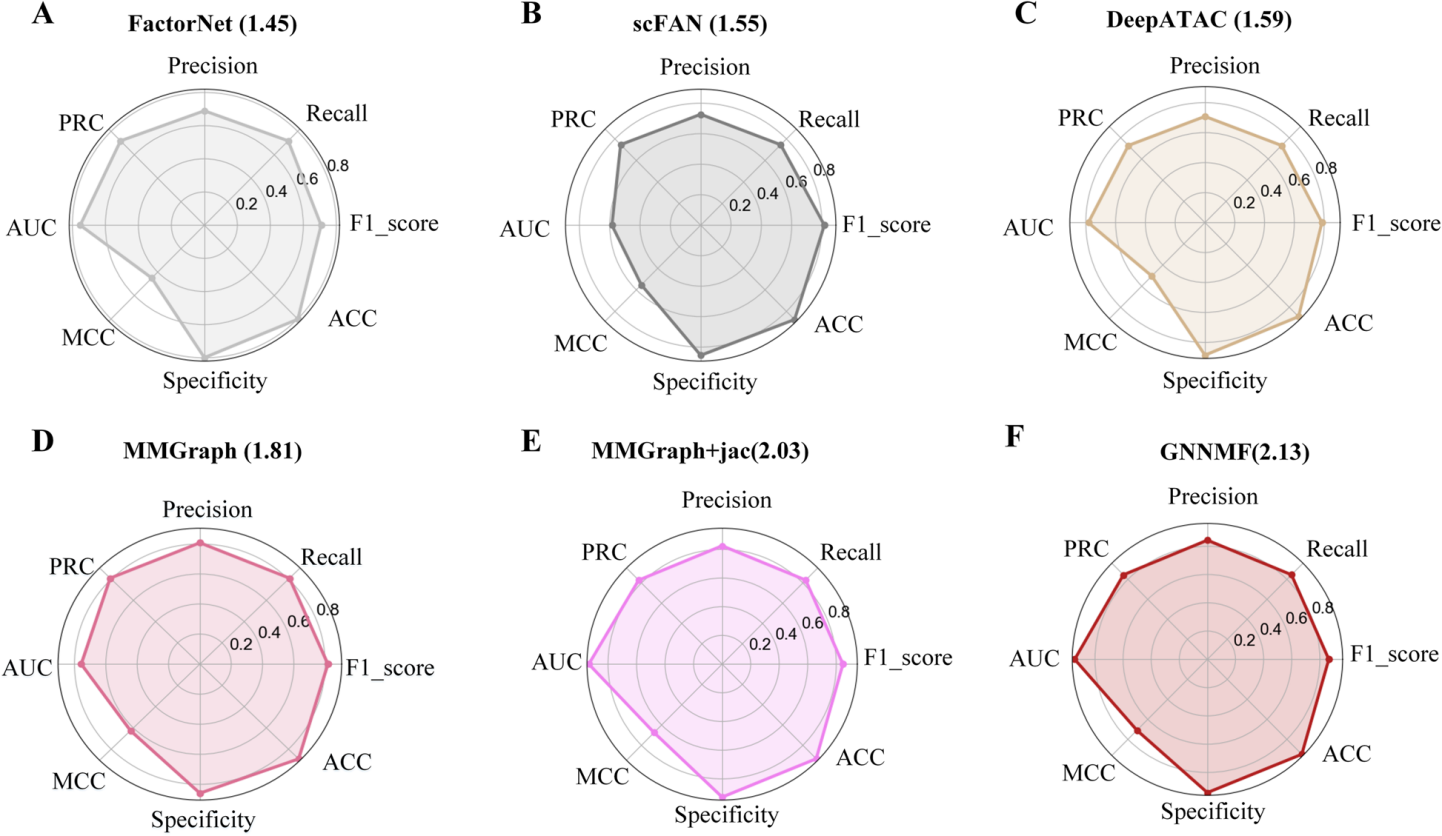
AEMRs of six models on 200 human ATAC-seq datasets

In this study, we utilized nine metrics to evaluate the performance of all the models on 200 human ATAC-seq datasets and 80 mouse ATAC-seq datasets (Fig. 3). FactorNeT, scFAN, DeepATAC, MMGraph, and MMGraph+jac were selected as comparison tools, because they achieved feasible performances in the previous study. In the light of results, AEMRs of models based on the GNN model are higher than those of the models based on the CNN model, which demonstrates that the GNN model is more suitable for ATAC-seq TFBS prediction. On 200 human ATAC-seq datasets, GNNMF achieved the highest average AEMR score (2.13). Compared to existing models, GNNMF improved the AEMR by 4.92%. Among models based on CNNs, DeepATAC obtained an AEMR of 1.59 , which is higher than those of scFAN and FactorNeT. On 80 mouse ATAC-seq datasets, GNNMF achieved consistent results on 200 human ATAC-seq datasets, and improved the AEMR by 6.81% (Supplementary Figure S1). On 280 ATAC-seq datasets, GNNMF yielded the highest AEMR score, which indicated that our improvement was efficient.

On 200 human ATAC-seq datasets, GNNMF obtained the highest scores for all eight metrics and achieved average precision, recall, F1_score, ACC, specificity (Supplementary Figure S2), MCC, AUC, and PRC values of 0.863, 0.846, 0.843, 0.845, 0.944, 0.709, 0.942, and 0.949, respectively. In particular, GNNMF improved the ACC and MCC by 2.13% and 5.08%, respectively. Moreover, on 80 mouse ATAC-seq datasets, GNNMF achieved average precision, recall, F1_score, ACC, specificity, MCC, AUC, and PRC values of 0.844, 0.831, 0.828, 0.830, 0.917, 0.676, 0.931, and 0.946, respectively (Supplementary Table S3). Compared to existing models, GNNMF improved the ACC and MCC by 2.36% and 6.35%, respectively.

### Finding multiple ATAC-seq motifs

**Fig. 4:**
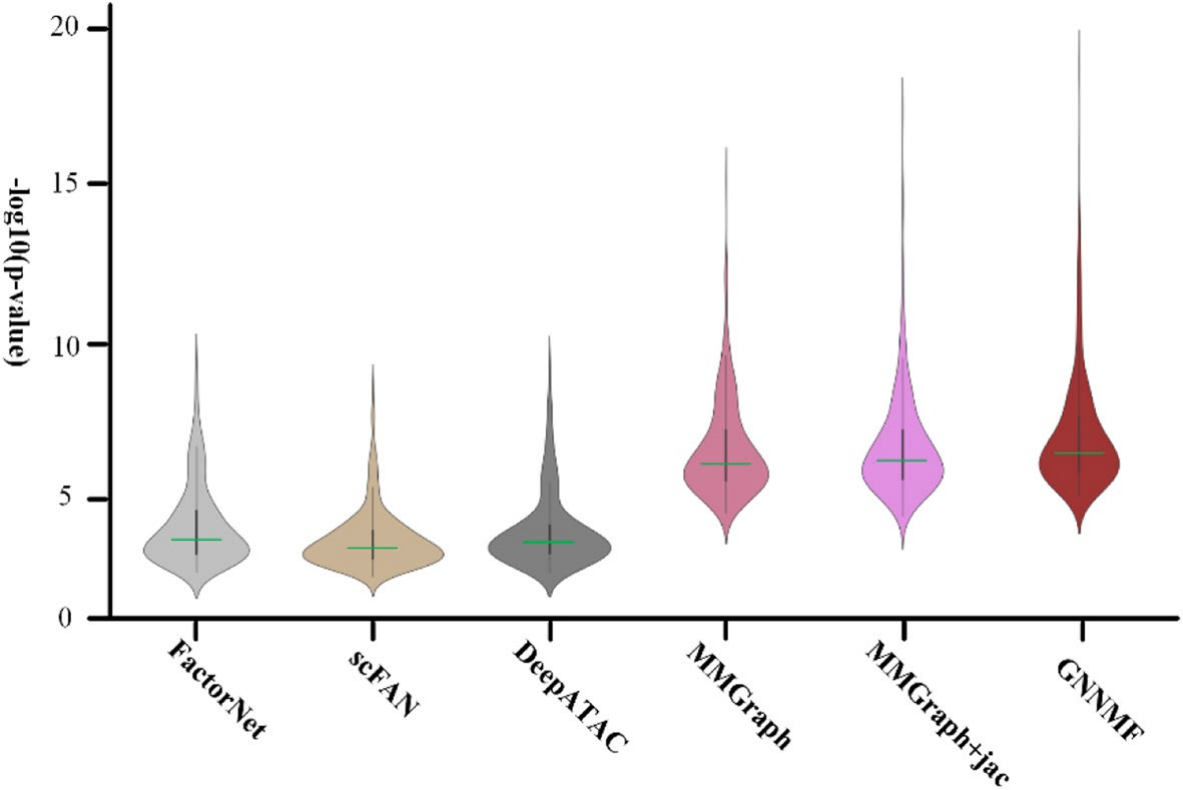
*p*-values of the found motifs of six models on 200 human ATAC-seq datasets

ATAC-seq can reveal all opening chromatin, which means that there are multiple motifs in an ATAC-seq dataset. In this study, we developed the GNNMF model to find ATAC-seq motifs by employing the GNN and coexisting probability. Existing DL models to find ATAC-seq motifs, including FactorNet, scFAN, DeepATAC, and MMGraph, were selecteds as comparison tools. We tested the above models on 280 ATAC-seq datasets including 200 human ATAC-seq datasets and 80 mouse ATAC-seq datasets. This study matches the found motifs to the HOCOMOCO database via the TOMTOM v5.1.0 tool. The *p*-value less than 0.05 indicated that the found motifs are significant. All motifs that each model found are listed in Table 1, 401 human ATAC-seq motifs were found. Among all the models, GNNMF found the most ATAC-seq motifs. Moreover, we tested all the models on 80 mouse ATAC-seq datasets, and the number of motifs in which each model found is listed in Supplementary Table S4. The *p*-value depicts the degree to which the found motifs are significant. The *p*-values of the found motifs of each model are shown in the violin plot, and the median value shows the model’s ability to find ATAC-seq motifs (Fig. 4). GNNMF achieved the highest median value among all the models. Based on motif finding results, GNNMF found the most motifs among all the models. The GNN-based models outperformed the CNN-based models, which indicated that the GNN is a feasible algorithm for finding ATAC-seq motifs. GNNMF is based on MMGraph, which is improved via the Jaccard edge, background probability, and the iterloss function. Our results demonstrated that our improvements were conducive to finding ATAC-seq motifs.

**Table 1:** Count of motifs that each model found on 200 human ATAC-seq datasets

**Models**	**FactorNet**	**scFAN**	**DeepATAC**	**MMGraph**	**MMGraph+jac**	**GNNMF**
**Motifs.no**	270	291	281	375	375	385

## Discussion

This study developed a GNN model called GNNMF by improving the MMGraph model. We improved the MMGraph by using the Jaccard edge, iterloss function, and background probability. GNNMF decomposed the multi-view heterogeneous graph into four subgraphs, and employed a three-layer GNN model to predict TFBSs. The first layer of GNNMF was used to learn the embedding of k-mer nodes via the similarity subgraph, Jaccard subgraph, and coexisting subgraph. The second layer was used for the embedding of sequence nodes via the inclusive subgraph, and the third layer was used to predict TFBSs. GNNMF utilizes embeddings of k-mer nodes and sequence nodes to define k-mer seeds and employs the coexisting probability to find multiple motifs with different lengths. Existing models to find ATAC-seq motifs, including FactorNet, scFAN, DeepATAC, MMGraph and MMGraph +jac, were selected as comparison tools. We tested all the models on 200 human ATAC-seq datasets and 80 mouse ATAC-seq datasets. We utilized the precision, recall, F1_score, specificity, ACC, MCC, AUC, and PRC to evaluate the models’ ability in TFBS prediction. In light of our results, GNNMF achieved the highest precision, recall, F1_score, ACC, specificity, MCC, AUC, and PRC of 0.863, 0.846, 0.843, 0.845, 0.944, 0.709, 0.942, and 0.949, respectively, on 200 human datasets. On 88 mouse datasets, our proposed model obtained the highest precision, recall, F1_score, ACC, specificity, MCC, AUC, and PRC values of 0.844, 0.831, 0.828, 0.830, 0.917, 0.676, 0.931, and 0.946, respectively. Moreover, the GNN-based model achieved higher AEMR scores than models based on CNN. Our results demonstrated that the GNN has more potential for TFBS prediction on ATAC-seq datasets. Compared to MMGraph+jac, GNNMF utilizes the iterloss to predict TFBSs. The AEMR of GNNMF was higher than that of MMGraph+jac, which indicated the iterloss has an advantage over BCEloss in TFBS prediction.

GNNMF utilized the embedding of k-mer nodes and sequence nodes, and calculated coexisting probability between k-mer nodes to find multiple motifs with different lengths. MMGraph and MMGraph+jac used the same way to set the background probability threshold, and they set the background probability threshold to 0.5. However, GNNMF defines the background probability threshold via negative sequences. Our results indicated that GNNMF found more and higher quality motifs than MMGraph and MMGraph+jac. Therefore, the background probability threshold of the negative sequences aided GNNMF in finding ATAC-seq motifs. FactorNet, scFAN, and DeepATAC are all based on CNNs, they found ATAC-seq motifs by convolutional kernels in the first layer. But these models only found the fixed length of motifs, and the found motifs are similar. However, compared with models based on CNNs, models based on GNNs have great advantages. We utilized all the models to find ATAC-seq motifs and used *p*-value to evaluate the motifs’ quality. The results demonstrated that GNNMF is the best model for finding multiple ATAC-seq motifs.

GNNMF employs a three-layer GNN to predict whether given sequences are bound by TFs. Some novel algorithms can be applied in TFBS prediction, such as graph attention networks [36], graphGAN [37], and graph autoencoders [38]. In the heterogeneous graph, there are many relationships between nodes and each kind of relationship represents the importance between two nodes. The attention mechanism can allocate and update the different weights to the nodes and edges of the heterogeneous graph, during the training process of the model. Moreover, TFs bind indirectly to motifs of other TFs, which co-regulate targeted gene expression [39]. The cooperation of TFs acts as a vital role in the process of human disease [40]. ATAC-seq data can detect open-accessible DNA regions by probing open chromatin, meaning that ATAC-seq data contain multiple TFs [41]. By analyzing ATAC-seq data, we revealed interactions between TFs, and explored the inducement of human disease. Therefore, GNNMF is a potential tool for studying the cooperation among different TFs.

## Conclusions

In this study, we developed a novel model named GNNMF for finding multiple ATAC-seq motifs. GNNMF built the multi-view heterogeneous graph by using ATAC-seq sequences, employed a three-layer of GNN to predict TFBSs, and utilized coexisting probability to find ATAC-seq motifs. We conducted experiments on 200 human and 80 mouse ATAC-seq datasets to analyze the effectiveness of the proposed method. Our evaluation metrics included precision, recall, F1_score, ACC, specificity, MCC, AUC, PRC, and AEMR. In particular, GNNMF improved the AEMR by 4.92% and 6.81% on 200 human and 80 mouse ATAC-seq datasets, respectively. Meanwhile, GNNMF found multiple motifs from ATAC-seq data through the coexisting probability between k-mers. Regarding ATAC-seq data, our proposed method found more and higher quality motifs, which demonstrated methods based on coexisting probability of k-mers are more efficient than DL models. As a result, GNNMF achieved better performance than a few state-of-the-art methods. This study made great contributions to finding motifs from ATAC-seq data.

### Supplementary Information

**Supplementary Material 1.** 

## Data Availability

The datasets are provided within Supplementary Table S1, which can be downloaded from the ENCODE project. GNNMF is available at https://github.com/zhangsq06/GNNMF.git.

## Abbreviations

ATAC-seq: Assay for Transposase-Accessible Chromatin using sequencing
TFBSs: transcription factor binding sites
DL: Deep learning
CNNs: convolutional neural networks
GNN: Graph neural network
TFs: Transcription factors
PWM: position weight matrix
ChIP-seq: Chromatin immunoprecipitation sequencing
DNase-seq: DNase I hypersensitive sites sequencing
AEMR: area of eight metrics radar
ACC: accuracy
MCC: Matthews correlation coefficient
AUC: Area under the receiver operating characteristic curve
TF-IDF: Term frequency-inverse document frequency
