# Supplementary material for: GNNMF: a multi-view graph neural network for ATAC-seq motif finding
Source: BMC Genomics. 2024 Mar 21;25:300. doi: 10.1186/s12864-024-10218-0 (PMC10956247; doi:10.1186/s12864-024-10218-0)
Supplement: Supplementary file 1 — Supplementary Material 1. [file 12864_2024_10218_MOESM1_ESM.docx]

**Supplemental materials for “GNNMF: A multi-view graph neural network for ATAC-seq motif finding”**

Shuangquan Zhang^1^, Xiaotian Wu^2^, Zhichao Lian^1*^, [Chunman Zuo](javascript:;)^3^ and Yan Wang^2,4*^

^1^School of Cyber Science and Engineering, Nanjing University of Science and Technology, Nanjing 210094, China.

^2^School of Artificial Intelligence, Jilin University, Changchun, 130012, China.

^3^Institute of Artificial Intelligence, Donghua University, Shanghai 201620, China.

^4^ Key Laboratory of Symbol Computation and Knowledge Engineering of Ministry of Education, College of Computer Science and Technology, Jilin University, Changchun, 130012, China.

**Table S1.** GSE number of 200 humans and 80 mouse ATAC-seq datasets

| **Human GSE number** | | | | |
| --- | --- | --- | --- | --- |
| GSE187422 | GSE187174 | GSE170876 | GSE172792 | GSE172929 |
| GSE170016 | GSE170354 | GSE173002 | GSE172603 | GSE187468 |
| GSE170146 | GSE187378 | GSE173068 | GSE172647 | GSE187697 |
| GSE172802 | GSE172592 | GSE172719 | GSE170378 | GSE172775 |
| GSE172620 | GSE169789 | GSE172793 | GSE169891 | GSE139692 |
| GSE172833 | GSE170318 | GSE169829 | GSE173044 | GSE170971 |
| GSE172886 | GSE188076 | GSE170676 | GSE188102 | GSE172966 |
| GSE172986 | GSE169767 | GSE172751 | GSE187858 | GSE170447 |
| GSE187710 | GSE170824 | GSE172991 | GSE172711 | GSE172607 |
| GSE172981 | GSE172593 | GSE170309 | GSE188147 | GSE172594 |
| GSE170848 | GSE172863 | GSE187201 | GSE170491 | GSE172805 |
| GSE170216 | GSE169772 | GSE172734 | GSE172855 | GSE169929 |
| GSE172783 | GSE187335 | GSE187298 | GSE172884 | GSE188165 |
| GSE173039 | GSE172883 | GSE187976 | GSE114204 | GSE172772 |
| GSE173000 | GSE170869 | GSE172786 | GSE172894 | GSE169798 |
| GSE187753 | GSE170616 | GSE172626 | GSE170604 | GSE170632 |
| GSE170195 | GSE187915 | GSE172674 | GSE172949 | GSE170721 |
| GSE187325 | GSE172788 | GSE170968 | GSE172869 | GSE172934 |
| GSE172747 | GSE172752 | GSE139801 | GSE172598 | GSE170039 |
| GSE170245 | GSE172797 | GSE170758 | GSE172595 | GSE172846 |
| GSE172727 | GSE187780 | GSE188057 | GSE170652 | GSE172977 |
| GSE169990 | GSE170433 | GSE172605 | GSE170251 | GSE172574 |
| GSE173031 | GSE172945 | GSE173067 | GSE170250 | GSE170518 |
| GSE172982 | GSE172975 | GSE170045 | GSE172826 | GSE187942 |
| GSE170765 | GSE187379 | GSE169879 | GSE172959 | GSE172559 |
| GSE187659 | GSE172699 | GSE170578 | GSE187329 | GSE172558 |
| GSE172702 | GSE173027 | GSE114205 | GSE172878 | GSE172954 |
| GSE172979 | GSE170321 | GSE169805 | GSE172700 | GSE172646 |
| GSE170747 | GSE170761 | GSE170909 | GSE170988 | GSE172790 |
| GSE169955 | GSE172741 | GSE187647 | GSE172563 | GSE170300 |
| GSE170012 | GSE170791 | GSE172952 | GSE172976 | GSE172635 |
| GSE170282 | GSE172970 | GSE170430 | GSE114201 | GSE173023 |
| GSE170214 | GSE172679 | GSE172904 | GSE172634 | GSE170959 |
| GSE172864 | GSE172760 | GSE170507 | GSE172803 | GSE172710 |
| GSE114202 | GSE187830 | GSE173043 | GSE169809 | GSE172685 |
| GSE172841 | GSE172538 | GSE172518 | GSE170176 | GSE187631 |
| GSE114200 | GSE139763 | GSE187043 | GSE172701 | GSE170918 |
| GSE187195 | GSE172546 | GSE187421 | GSE172722 | GSE170899 |
| GSE187937 | GSE172731 | GSE172765 | GSE172990 | GSE187646 |
| GSE187196 | GSE170014 | GSE170073 | GSE187312 | GSE187426 |

| **Mouse GSE number** | | | | |
| --- | --- | --- | --- | --- |
| GSE173064 | GSE172713 | GSE173015 | GSE172698 | GSE173052 |
| GSE172917 | GSE172767 | GSE172812 | GSE172659 | GSE172998 |
| GSE172688 | GSE172703 | GSE172813 | GSE172876 | GSE94167 |
| GSE172822 | GSE172694 | GSE172572 | GSE172935 | GSE172658 |
| GSE172667 | GSE172866 | GSE172763 | GSE173028 | GSE172759 |
| GSE172744 | GSE172836 | GSE173061 | GSE173059 | GSE172718 |
| GSE172852 | GSE172663 | GSE172911 | GSE173049 | GSE172997 |
| GSE172969 | GSE172657 | GSE172912 | GSE172784 | GSE173025 |
| GSE172861 | GSE172670 | GSE172561 | GSE172843 | GSE172845 |
| GSE172535 | GSE172993 | GSE172610 | GSE172738 | GSE172818 |
| GSE172887 | GSE172554 | GSE172900 | GSE172625 | GSE94212 |
| GSE173040 | GSE172531 | GSE172871 | GSE172630 | GSE173018 |
| GSE172562 | GSE172664 | GSE172576 | GSE172740 | GSE172874 |
| GSE94181 | GSE172837 | GSE172676 | GSE173036 | GSE172723 |
| GSE172627 | GSE172933 | GSE172547 | GSE172668 | GSE172597 |
| GSE172616 | GSE173047 | GSE172800 | GSE94226 | GSE173037 |


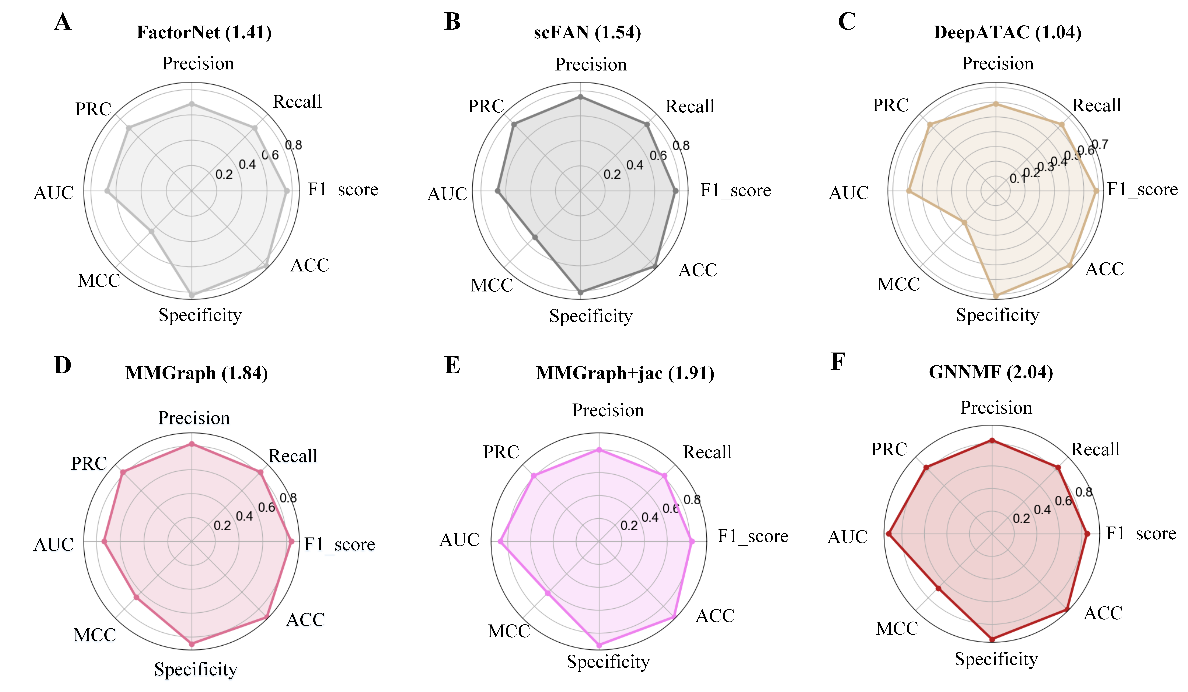


**Figure S1.** The eight matrices of six models on 80 mouse ATAC-seq datasets


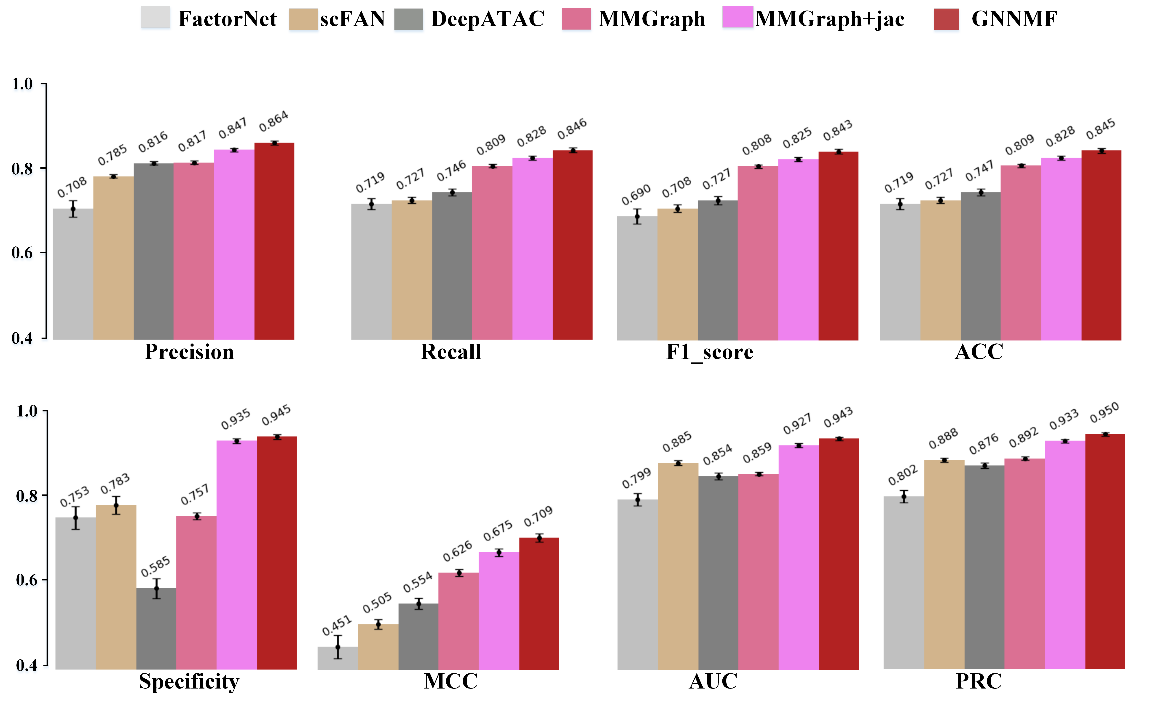


**Figure S2.** Eight matrices of all models on 200 human ATAC-seq datasets

**Table S2.** The hyper-parameters of GNNMF model

| Hyper-parameter | learning rate | *dsim*, *dco*, *djac* | *dsq* | *lenk* |
| --- | --- | --- | --- | --- |
| Value | 0.01, 0.001, 0.0001 | 50, 100, 150 | 100, 150, 200 | 3, 5, 7, 9 |

**Table S3.** Eight metrics scores of all models on 80 mouse ATAC-seq datasets

| MODELs | Precision | Recall | F1_score | ACC | Specificity | MCC | AUC | PRC |
| --- | --- | --- | --- | --- | --- | --- | --- | --- |
| FactorNet | 0.755 | 0.705 | 0.688 | 0.706 | 0.672 | 0.453 | 0.825 | 0.838 |
| DeepATAC | 0.683 | 0.636 | 0.588 | 0.635 | 0.591 | 0.301 | 0.709 | 0.713 |
| scFAN | 0.765 | 0.756 | 0.754 | 0.757 | 0.666 | 0.521 | 0.812 | 0.852 |
| MMGraph | 0.839 | 0.821 | 0.818 | 0.822 | 0.738 | 0.661 | 0.856 | 0.896 |
| MMGraph+jac | 0.814 | 0.812 | 0.802 | 0.811 | 0.867 | 0.635 | 0.903 | 0.931 |
| **GNNMF** | **0.844** | **0.831** | **0.828** | **0.830** | **0.917** | **0.676** | **0.931** | **0.946** |

**Table S4.** Count of motifs that each model found on 80 mouse ATAC-seq datasets

| Models | FactorNet | scFAN | DeepATAC | MMGraph | MMGraph+jac | **GNNMF** |
| --- | --- | --- | --- | --- | --- | --- |
| Motifs.no | 134 | 291 | 128 | 284 | 414 | **662** |


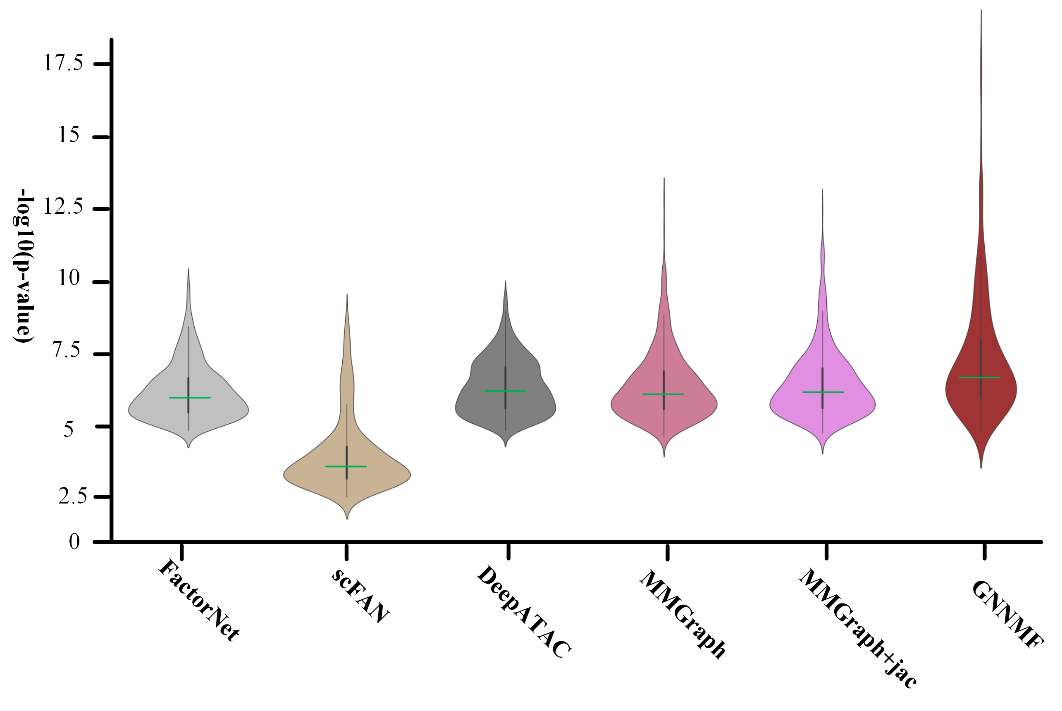


**Figure S3.** The p-values of found motifs of six models on 80 mouse ATAC-seq datasets
